# Some Observations upon Suppuration of the Maxillary Antrum; with Special Reference to Diagnosis and Treatment

**Published:** 1904-02

**Authors:** 


### Reviews of Dental Literature.

Some Observations upon Suppuration of the Maxillary Antrum; with Special Reference to Diagnosis and Treatment.[1] By Herbert Tilley, M.D., B.S. (Lond.), F.R.C.S. (Eng.).[2]

The natural opening of the antrum into the nose is situated in the membranous upper portion of the inner antral wall. Seen from within the nasal cavity, it will be noticed that it opens into the channel of the infundibulum in the middle meatus of the nose. The high situation of the opening renders it very unsatisfactory for purposes of drainage. (Diagrams were shown illustrating these points.)

I would ask you to particularly bear in mind two anatomical features which have an important bearing upon the pathology and treatment of nasal accessory sinus suppuration. The first is the relation of the maxillary antrum to the frontal sinus. It will be noticed that the infundibulum terminates at or in the antral opening, and that a fold of mucous membrane extends upward from the foramen, forming a pocket, at the bottom of which is the antral foramen, the fold referred to being on the inner side.

It results from this that a discharge issuing from the frontal sinus would tend to fill the antrum before the latter began to overflow into the nose, and hence the lower sinus may act as a reservoir for discharges from the ethmoidal cells or frontal sinus, without being itself primarily diseased.

Again, Tillaux has shown that if water be injected into the frontal sinus a considerable quantity of it flows into the cavity of the antrum. Hence, the fact that an antrum contains pus does not necessarily mean that it is produced there, for it may be merely acting as a reservoir rather than as a generator of the discharge. The following case will illustrate my meaning:

---

[1] Abstract of a paper read before the Odontological Society of Great Britain, November 23, 1903.

[2] Surgeon to the Hospital for Diseases of the Throat, Nose, and Ear, Golden Square.

Mr. F., aged fifty-four, had for five years suffered from a purulent nasal discharge, associated with nasal obstruction due to large polypi within the nasal cavities. On several occasions the polypi were removed, but the discharge continued as freely as before, and it was ascertained that it proceeded from the frontal, ethmoidal, sphenoidal, and antral sinuses. Both maxillary antra were drained by the alveolar method, and for two years were irrigated twice daily with antiseptic lotions. The purulent discharge, although lessened in amount and robbed of its fetor, continued to flow, and the patient used on an average fifty handkerchiefs a week. He finally decided to submit to radical operations upon the different sinuses, with a view to the discharge being entirely cured.

Last June I operated upon both frontal sinuses, and to my astonishment found that it was not necessary to further treat the antra, for from the day on which the operation on the higher sinuses was performed not a single drop of pus could be washed from the antra. Surely it is a remarkable thing that these sinuses (antra) could have merely acted as reservoirs of pus for so long a time without themselves becoming actual generators of the same.

The last anatomical feature to which I would like to draw your attention is that sometimes, as is shown in the diagram, some of the lower anterior ethmoidal cells spread outward in the bony floor of the orbit, and infection may spread from these into the antrum. It is of the utmost importance in the radical operation for the cure of chronic suppuration to see that these cells (if they exist) are not overlooked; for if left behind in a septic condition they would reinfect the antral cavity.

*Causes of Antral Suppuration.*—As many of you are aware, it used to be thought that all cases of antral suppuration were caused by diseased teeth, but during recent years it has become an equally well-established fact that many cases arise by infection from the nose, and this is especially likely to occur during the course of one of the acute specific fevers. Influenza has proved a prolific parent of suppuration within the nasal accessory sinuses, while in a smaller number of cases erysipelas, scarlet fever, measles, diphtheria, typhoid, and pneumonia have been definitely proved to be the cause of the infection. A certain amount of catarrh of the nasal mucosa is frequently present during the course of these diseases, and since the nasal mucous membrane is continuous with that lining the accessory cavities, the latter are very prone to become affected by simple extension of the catarrhal process. Hence we can easily understand that an acute nasal catarrh may be followed by a similar condition, with retention of the secretions in the accessory sinuses, a condition possibly accounting for some of the frontal discomfort experienced during an acute "cold in the head." If owing to inefficient drainage such secretions be retained under tension, an increase of inflammation may result, and should certain micro-organisms gain access to the suitable medium thus provided, suppuration may occur—it may be in the antrum, in a single ethmoidal cell, in a frontal sinus, or in any combination of these cavities.

If we suppose an ethmoidal cell to be affected, and to have become the focus of suppuration, it is at least possible that the contained pus may find its way into the maxillary antrum, or even into a frontal sinus, and *vice versa.* So that—given an acute or chronic catarrhal condition of the mucous membrane acting as a predisposing cause—the exciting cause of empyema may be organisms associated with influenza, syphilitic nasal lesions, insanitary surroundings, or convalescence from long illnesses, especially acute infectious diseases; while, as will hereafter be stated, most cases of antral suppuration are due to septic infection starting from the root of a diseased tooth, especially the second bicuspid or one of the molar teeth.

Traumatisms will account for a certain number of cases,—possibly more than we are inclined to admit,—and I fear that the dental as well as the nasal surgeon is not always free from blame. For example, I have a vivid recollection of three or four cases where symptoms of empyema have immediately followed the attempt at extraction of a tooth, in which the history would seem to suggest that a portion of a dental root was broken into the antrum. Or again, the symptoms appeared shortly after a tooth had been "filled," and were preceded by intense toothache, followed by a sudden relief which was associated with a foul smell within and a purulent discharge from the nose. Under such circumstances it is probable that the cavity within the tooth had not been rendered perfectly aseptic before the "filling" had been inserted, and the resulting suppuration had followed the path of least resistance and found its way into the overlying sinus.

On the other hand, I have known antral suppuration to follow upon the careless use of the galvano-cautery in the middle meatal region of the nose; and in two other instances it complicated the convalescence of an operation undertaken for the removal of an outgrowth from the nasal septum. Under the latter circumstances, when the nasal mucosa are in an irritable condition, and septic accumulations lie within the nasal cavities, it is particularly easy for a patient in blowing the nose to force such material into the neighboring sinuses and thus to start a chronic suppurative condition.

The question, however, which I feel sure many of you will ask me will be, What proportion of cases of chronic antral suppuration do you consider arises from dental causes?

I am afraid it is impossible for me to give you anything like a direct answer, but this statement may interest you,—namely, that with one exception, in each of the sixty-four cases upon which this paper is based, diseased bicuspid or molar teeth were present (or by their absence implied previous removal for disease) upon the side corresponding to the antral suppuration. I would even go farther, and say that during the past ten years, during which I must have seen at least three hundred cases of "antral abscess," I have only met with one patient (a girl, aged twelve) in whom the teeth were quite healthy.

These are somewhat startling facts, but I am able to substantiate them, even though there rings in my ear the statement of Grünwald, of Münich, that, out of ninety-eight cases of antral suppuration, in fourteen only could he trace with certainty their origin from diseased teeth. How does this curious difference in large experiences arise? Possibly in this way. You will notice that in my statement it is asserted that "diseased" teeth were present in all cases, and by "disease" is meant any departure from the normal, ranging from a tiny carious cavity in the crown of a tooth to a septic condition involving the crown, roots, and surrounding alveolar sockets. In a few of my cases it was difficult to believe that the antral suppuration had been influenced in any way by a small carious cavity in the crown of what (with this exception) was a sound tooth, and it is probably all such mild cases of dental disease which Grünwald excluded from his statistics. Nevertheless, the constancy of the association between the diseased teeth and the sinus suppuration would seem to me to be more than mere coincidence.

Or can we explain the facts in this way? That in this country there are very few people beyond the age of puberty whose teeth are absolutely sound, and therefore the chances of any one individual who is suffering from chronic antral suppuration (relatively a much rarer condition) having unhealthy teeth on the same side are very great.

Or again, may we not reasonably suppose that a small amount of disease, limited even to the crown of a tooth, will probably set up a certain degree of irritability of the mucous membrane of the antrum in the neighborhood of the root of that tooth—shall we say an increased vulnerability, which renders it more liable to infection from the nose? Unless it be assumed that my figures are the result of mere coincidence, I fear we must adopt some such explanation as this, and in this connection would quote what Grünwald says, in spite of his opposition to the frequency of dental disease as a cause of antral suppuration. He says, "I would especially emphasize the fact that a tooth must not be considered harmless because the socket is not diseased. Infection creeps along the lymphatics of healthy bone, and a focus of infection in the crown of a tooth is by no means to be despised, for even if an empyema of the antrum be due to another cause, yet disease of the crown of a tooth is calculated to maintain such a state of irritation in the mucous membrane as may frustrate all attempts at cure."

*Symptoms.*—The symptoms of acute antral suppuration are so well known to you that I need scarcely do more than mention the acute throbbing pain in the cheek and supraorbital region, tenderness of one or more teeth and their immediate neighborhood, especially that of the canine fossa. Sometimes the soft tissues of the face and cheek are also swollen and tender, while fever and the constitutional symptoms associated with it add to the sufferings of the patient. Relief comes when the abscess bursts into the nose, or is relieved by extraction of the inflamed tooth and (if necessary) perforation through the alveolar socket.

A patient suffering from chronic antral suppuration will, I take it, consult the dental surgeon only when dental symptoms are prominent, but the medical attendant is often appealed to for the relief of symptoms which do not at first sight suggest the antrum; *e.g.*, "an offensive discharge from the nose," a frequently recurring "disagreeable taste," "increasing difficulty in breathing through the nose," due to the formation of polypi, "chronic nasal catarrh," "headache," "brow ague," feelings of "weight over the forehead," or "round the eyes." In other cases the digestion is impaired owing to more or less severe forms of gastritis, brought about by the swallowing of pus into the stomach; while absorption of the purulent material into the general circulation has a very subtle effect on the nervous system, inducing a lack of energy and general condition of depression, which may perhaps be best exemplified by the following passage taken from a patient's letter two months after the antra had been drained. She says, " Before the operation I was always depressed, and often cried several times a day; if I walked a mile I was tired out, but now I can walk eight miles without any fatigue whatever."

If the dental symptoms be associated with a " discharge of offensive matter from the nose," coupled with a " sickly taste" in the mouth, and at the same time there are frequent attacks of supra-orbital headache,—possibly of greater severity during the earlier hours of the day,—especially if these symptoms should be associated with any of those I have already mentioned, under such circumstances you may reasonably entertain a strong suspicion that the antrum is diseased, either alone or in combination with the other accessory sinuses.

*Diagnosis.*—Here, again, we must not dwell upon fine details, because, in order to recognize the chief diagnostic features of chronic antral suppuration as seen within the nose, I should have to presume that you had an intimate acquaintance with the appearances of the nasal cavities both in health and disease. Still less is it necessary for me to discuss the diagnosis of those difficult cases in which more than one of the accessory cavities are simultaneously affected. Two tests, however, which you may often apply with advantage are the following:

(1) Ask the patient to blow his nose thoroughly upon the affected side until no pus is expelled. Then let him rest for from three to five minutes with the suspected antrum uppermost, during which time the pus will probably flow into the nose. On again blowing the nostril, the yellow and often offensive discharge may once more be seen upon the handkerchief.

(2) Let the patient place the feet close together and endeavor to touch his toes without bending the knees. If this position be maintained for from one to two minutes, considerable congestion of the head will be caused, and aching of the inflamed tooth or diseased antrum, or corresponding frontal region, may be induced.

As I stated just now, the appearance of pus in the middle meatus of the nose, the presence of polypoid granulations or hypertrophies in this position, or the well-known swelling of the mucosa covering the uncinate process of the ethmoid ("Kaufmann's cushion"), cannot be discussed with any advantage upon this occasion.

Presuming we are dealing with an uncomplicated case of unilateral suppuration, there is one test which, whilst not by any means infallible, may give you strong confirmation of your suspicions of antral mischief, while it is painless and usually of considerable interest to the patient. I refer to transillumination by means of a small six- to ten-volt electric lamp placed within the patient's mouth, while the room is darkened or the head covered with a dark cloth, in order to obtain the fullest contrast between the lights and shades provided by the test.

If there be pus within the antrum, it will be noticed that there is no infraorbital "light crescent" upon the diseased side, or a much less definite one than on the healthy side. This infraorbital opacity is due, in my opinion, to a chronic inflammatory process in the bony walls of the antrum, because not only is it present immediately after the pus has been washed out, but may sometimes be seen forty-eight hours after the radical operation has been performed, and when the sinus cavity is without any membranous lining at all. Unfortunately the test is not always reliable because a similar opacity may be noted in healthy but thick-walled antra, and of course its value is diminished when both sinuses are diseased. Other conditions may also modify its reliability, but in the great majority of cases it may very materially assist you in *confirming suspicions* founded upon other symptoms presented by the case.

(A practical illustration of transillumination in a patient suffering from chronic antral suppuration was given.)

There is only one absolutely certain means by which you can demonstrate the presence or absence of pus in the antrum, and that is by exploration of the cavity. Formerly it was (and I fear, in many quarters still is) the custom to perforate the alveolus under nitrous oxide anæsthesia and, if necessary, to remove a tooth for the purpose. Should the exploration demonstrate the absence of pus, the patient will possibly have lost a useful tooth, to say nothing of the inconvenience of the anæsthetic, and of—not improbably—a considerable amount of after-pain and discomfort in the jaw.

All these disadvantages can be obviated by making a puncture within the nose. This little operation is practically painless. It needs no general anæsthetic, it may be done in the consulting-room, and its evidence is absolutely reliable.

A small dossil of wool is moistened with a ten per cent. solution of cocaine and applied by means of a probe to the inner wall of the antrum underneath the anterior end of the inferior turbinal bone. In a few moments a (Lichtwitz's) fine trochar and canula are passed outward, backward, and slightly upward, through the inner antral wall. The trochar is withdrawn, leaving the canula in position, and to its proximal end is fitted a rubber tube through which some warm boracic or normal saline solution can be injected. If there be any pus in the sinus it is at once demonstrated by this simple, bloodless, and absolutely reliable method. Curiously enough, in a number of cases in which I have used it, immediately the antrum had been perforated the patient at once complained of aching in one of the diseased teeth upon the same side. This was very valuable information, for it at once suggested which one, of perhaps several unsound teeth, was the real cause of the trouble.

*Treatment.*—The treatment of acute antral suppuration need not detain us long. The painful symptoms rapidly subside if free drainage be provided, either through a tooth socket in the alveolus, or through a large opening in the inferior meatus of the nose. If the antrum be irrigated daily with some mild antiseptic for ten days to a fortnight, the suppuration will cease and the opening in the alveolus may be allowed to close. Very occasionally one irrigation will suffice and the insertion of a drainage-tube will be unnecessary. It is a moot question whether any tube is necessary in acute cases or whether they will not do better if only a moderate-sized alveolar perforation be made. Personally, I prefer a tube, because we can be sure of efficient drainage until it is certain that suppuration has ceased, whereas, should the opening close before the discharge has quite disappeared, it might be necessary to open up the alveolar perforation for a second time.

With regard to chronic suppuration, you will gather from what has already been stated that in the treatment of every case the first desideratum is that the teeth be attended to, and this even when the history may seem to indicate that the primary infection entered by way of the nose. However clear such a sequence may be, a diseased tooth may add such irritative factors as to rob any other methods of treatment of complete success, and hence the reason why your services should be requisitioned.

Next will come the question, What form of local treatment shall be adopted for checking the suppuration within the antrum? Will you pardon me if I presume to remind you that we are dealing with a bony-walled cavity which is lined by a pyogenic membrane, and that our first endeavor should be to restore the latter to its natural condition, or, failing this, we must adopt other means to cure the patient's symptoms. In any case we must be guided by those well-known principles of surgery which are adopted in other regions of the body in the treatment of abscess cavities. I would not emphasize this point had my experience not amply proved to me that such principles are often more honored in the breach than in the observance. Now there are three principles which should influence us in the treatment of all our cases:

(1) *Efficient drainage,* the ultimate success of which will be accelerated by frequent irrigations of the unhealthy mucous membrane with mild antiseptic washes.

(2) *More or less obliteration of the diseased cavity.* This last method will be the final one if failure attends a fair or lengthy trial of simpler measures.

(3) *Attention to the general health of the patient.* We must never forget that defective hygienic surroundings and excesses in eating, drinking, and smoking have a very great influence for evil upon the general progress of the case. Immoderate use of alcohol or tobacco have a great tendency to induce congestion and chronic catarrhal conditions of the mucosa of the upper air-passages, conditions which are very adverse to rapid recovery after operative interference in these regions. Occasionally two or three grains of calomel over night, followed in the morning by a saline draught, will produce more improvement than all those new antiseptics of "high destructive power," the advertisements of which form an increasing constituent of our waste-paper baskets.

*Drainage* (alveolar method).—Since efficient drainage can only take place when our opening is situated at the lowest level of the abscess cavity, it need scarcely be said that an alveolar perforation is better than drainage through the canine fossa, or through the inferior meatus of the nose. Drainage through the canine fossa is inefficient, and the tube or plug often causes great irritation of the mucous membrane of the cheek or gums, while any but a large opening in the inferior nasal meatus has a very great tendency to close, in addition to which the patient nearly always finds a difficulty in carrying out the necessary irrigations.

You all know the many and ingenious alveolar drainage-tubes which from time to time have been invented. Those seem to answer best in which a plate fixed to adjacent teeth supports a metal tube, the lumen of which is about the size of a crow-quill, and which is occupied by a split plug which can be inserted during meal-times in order to prevent access of food to the antrum. I have also seen cases do equally well in which a solid plug takes the place of the tube. Whichever form of tube you adopt, be careful that its upper end, when it is in position, does not stand high above the level of the antral floor, under which circumstances its very function as a drain will be destroyed.

As to the irrigating lotion which the patient shall use, I can only say that I am wedded to no one in particular, and am certain that the best results will be attained by constantly changing the nature of the antiseptic. A saturated solution of boracic acid, chlorate of potash (twenty grains to the ounce), carbolic lotion (1 in 60, for the early treatment of offensive cases), lysoform, the active principle of which is formalin, in strength five minims to a tumblerful of water, peroxide of hydrogen, ten per cent. solution, sulphate of zinc (two grains to one ounce), and finally normal saline solution,—all these may be found useful. In the later stages, after washing out the antrum, it may be well to inject and leave within the cavity one-half drachm of an alcoholic solution of boracic acid (pulv. ac. boracic, ten grains; spir. vini rect., aq. destill., of each, two drachms).

At the outset the patient should wash out the antrum twice daily; as the discharge lessens, once daily; with further improvement, every second or third day; and finally, if after an interval of ten days no pus returns upon irrigating, then the case may be considered cured and the tube removed.

Remember: (*a*) Always use the solution warm; (*b*) never use warm water alone, even when sterilized. Its density is different from that of blood serum, and will often induce a free discharge of mucus, and sometimes cause pain.

Three questions now suggest themselves:

(1) What is the result of such treatment?

(2) How long should it be continued?

(3) To what class of case should it be applied?

(1) With regard to the result, my experience is that in nearly all cases—even those of long duration—alveolar irrigation rapidly diminishes the amount and fetor of the pus, together with those more general symptoms which have been described; but that in cases of more than a few months' duration it is extremely difficult to get rid of the last trace of discharge, so that you can feel justified in removing the tube altogether. I have recently ascertained that out of twenty-seven of my patients in whom the alveolar treatment was adopted and irrigation patiently persevered in, only five have been able to give up their tubes. The rest still find a small amount of purulent secretion comes away on irrigation, and if they fail to carry on the treatment an increase in the amount of discharge is rapidly noticed.

(2) How long should the treatment be continued? Until the discharge entirely ceases, or until the failure of simple measures renders it obvious that, if the patient desires an absolute cure, some further and more radical treatment will be necessary. Out of my twenty-seven alveolar cases seen during the past two years, fifteen of them have worn the tube and carefully irrigated for at least six months. One patient had been doing so for ten years, another for three and a half, and a third for two years. Many of them found the tube caused no inconvenience, and preferred to go on with the irrigation. Five, however, elected to have the radical operation, and I am glad to say that they have without exception been cured, and by the term "cure" I mean that not a trace of pus can be found within the nose.

(3) To what class of cases should the alveolar treatment be applied? Certainly to all those the duration of which has been a matter of months rather than of years, and possibly as a first measure in most of the chronic cases, because now and again, even in these, alveolar drainage and irrigation have resulted in a rapid cure.

The advantages of the method are its simplicity, and in this respect it is scarcely a more serious operation than the removal of a tooth. The after-treatment can soon be carried out for long periods by the patient himself, and the general improvement which is effected is very great, even if the measure be not an entirely curative one. To the very old, the broken in health, the nervous, the busy man with the cares of a family and an exacting occupation, and to whom a week or ten days' lying up is a very serious matter, the alveolar method has many advantages. On the other hand, we meet with cases of long-standing suppuration, in which—no matter how persistently or with what variety the irrigating lotions are used—the discharge of pus from the nose into the mouth is still profuse, the patient becomes tired of irrigating, and wants to know "if something cannot be done to cure him once and for all?" Or, perhaps, he or she will be one of those impatient individuals who "do not want to be bothered with tubes," but fret day by day because they "can blow matter from the nose, and always have a nasty taste in the mouth." For this class of patients we may adopt other and more radical measures.

*The Radical Operation.*—An aseptic sponge having been passed into the nasopharynx and a second one placed between the cheek and teeth on the affected side, in order to prevent any flow of blood into the larynx, the anterior end of the inferior turbinal bone upon the diseased side is removed by a pair of angular scissors and a wire snare. An incision is then made in the gingivo-labial groove and over the canine fossa parallel to the alveolar process of the jaw, and extending from the malar process of the superior maxilla to the canine ridge. The soft parts and periosteum are turned up and the anterior wall of the antrum, as represented by the canine fossa, is removed by gouge and mallet. An opening is made in the canine fossa, between the size of a sixpence and a shilling, the larger within reason the better, but care should be taken not to wound the infraorbital nerve. The diseased mucous membrane is then carefully and thoroughly curetted away, the free hemorrhage which interrupts the procedure being checked by means of a good supply of sterilized strips of gauze. The same end may be more rapidly attained if, after having curetted away most of the diseased membrane, one or two strips of gauze be moistened in adrenalin chloride, or hydrogen peroxide solution, and tightly packed into the sinus and allowed to remain for from three to five minutes. The time thus lost will be more than regained by the greater ease with which subsequent proceedings can be carried out.

At this stage we have to decide how much of the inner antral wall shall be taken away in order to provide free drainage from the sinus into the nose. The following considerations should guide you. If the diseased mucous membrane be mainly confined to the lower half of the antrum and the middle meatal region of the nose be healthy, it will suffice if a counter-opening be made in the lower anterior region of the naso-antral wall; this opening should at least be as large as a sixpence because of the great tendency to cicatrization which characterizes wounds in this neighborhood.

If, on the other hand, the whole of the lining membrane be diseased, and especially if polypi or mucous membrane hypertrophies be seen in the middle meatus of the nose, the whole of the inner antral wall should be removed, and it is this modification of the simpler operation which has yielded me my best results. The reasons for this extensive ablation are twofold,—the lower half being removed for drainage purposes, the upper half in order to destroy that membranous portion of the inner wall which is often in a polypoid condition, and also to gain access to any lower ethmoidal or maxillo-ethmoidal cells which are so frequently diseased, and which, if left untouched, will reinfect the antrum. The sinus cavity is finally mopped out with strips of gauze soaked in carbolic lotion (1 in 20), and the operation is completed. It is unnecessary to insert any packing unless the hemorrhage be unusually free; in any case only a loose strip of gauze will be necessary, which should be removed forty-eight hours after the operation and not replaced. In a straightforward case the operation may be completed in from thirty to thirty-five minutes.

The after-treatment consists in douching out the nose, and by this means also the antrum, twice daily for two or three weeks with some mild antiseptic wash. The patient can sit up on the third day after the operation, and may usually go out within ten days. The bucco-antral wound heals very quickly (seven to ten days), and no deformity or falling in of the cheek occurs. I would particularly emphasize the latter point, because one of the objections which have been raised against the radical operation is the deformity which may result. During the past two years I have performed this operation thirty-seven times, and have kept myself intimately acquainted with the progress of each patient, and it is with no small feeling of satisfacton I can record thirty-four successful cases as against three imperfect results (*vide* "Synopsis of Cases"). The three patients to whom I refer were not operated upon by quite the same method as I have just detailed, and therefore cannot be said to detract from the value of the operation which I am advocating. The failure to produce complete cure in the patients referred to was due, I think, to the fact that no counter-opening was made into the nose.

Excepting for a troublesome neuralgia, which occurred in four cases and lasted for from four to ten days, I have met with no complication.

To the question, "What circumstances would guide you in advising a radical operation without preliminary trial of simpler measures?" we may answer, "The appearances presented by the middle meatus of the nose." If in a long-standing case this region be filled with polypoid granulations or swollen and œdematous mucous membrane, then it is fairly certain that the antral mucous membrane is also in an advanced state of chronic degeneration, and nothing short of radical treatment will effect a permanent cure.

You may quite naturally ask, "What is the condition of the antrum in a successful case, six months after operation?"

There can be no doubt that it is partially obliterated by the granulations which spring up over the bony walls uniting with those which grow inward from the soft tissues of the cheek through the large opening in the canine fossa. This mass of granulation tissue becomes covered with epithelium which spreads inward from the circumference of the naso-antral opening, because without this natural method of healing suppuration would inevitably continue.

It is only necessary to examine a patient four or five months after operation with a small mirror passed within the nose, or by means of a curved probe, to satisfy one's self that the original antral sinus is partially obliterated and is only represented by a concavity upon the outer side of the nasal fossa, which is very much smaller than the original antrum.

